# Hinokitiol-iron complex is a ferroptosis inducer to inhibit triple-negative breast tumor growth

**DOI:** 10.1186/s13578-023-01044-0

**Published:** 2023-05-13

**Authors:** Hongting Zhao, Meng Zhang, Jinghua Zhang, Zichen Sun, Wenxin Zhang, Weichen Dong, Chen Cheng, Yongzhong Yao, Kuanyu Li

**Affiliations:** 1 State Key Laboratory of Pharmaceutical Biotechnology, Jiangsu Key Laboratory of Molecular Medicine, Medical School, Nanjing University, 22 Hankou Road, Nanjing, 210093 China; 2 Department of General Surgery, Nanjing Drum Tower Hospital Clinical College of Nanjing Medical University, Nanjing, 210008 China

**Keywords:** Ferroptosis inducer, Iron, Hinokitiol, Triple-negative breast cancer, Lipid peroxidation

## Abstract

**Background:**

Ferroptosis is a unique cell death, dependent on iron and phospholipid peroxidation, involved in massive processes of physiopathology. Tremendous attention has been caught in oncology, particularly for those therapy-resistant cancers in the mesenchymal state prone to metastasis due to their exquisite vulnerability to ferroptosis. Therefore, a therapeutical ferroptosis inducer is now underway to be exploited.

**Results:**

A natural compound, hinokitiol (hino), has been considered to be an iron chelator. We have a novel finding that hino complexed with iron to form Fe(hino)_3_ can function as a ferroptosis inducer in vitro. The efficiency, compared with the same concentration of iron, increases nearly 1000 folds. Other iron chelators, ferroptosis inhibitors, or antioxidants can inhibit Fe(hino)_3_-induced ferroptosis. The complex Fe(hino)_3_ efficacy is further confirmed in orthotopic triple-negative breast cancer (TNBC) tumor models that Fe(hino)_3_ significantly boosted lipid peroxidation to induce ferroptosis and significantly reduced the sizes of TNBC cell-derived tumors. The drug’s safety was also evaluated, and no detrimental side effects were found with the tested dosage.

**Conclusions:**

When entering cells, the chelated iron by hinokitiol as a complex Fe(hino)_3_ is proposed to be redox-active to vigorously promote the production of free radicals via the Fenton reaction. Thus, Fe(hino)_3_ is a ferroptosis inducer and, therapeutically, exhibits anti-TNBC activity.

**Supplementary Information:**

The online version contains supplementary material available at 10.1186/s13578-023-01044-0.

## Background

Breast cancer is the most common malignant tumor and the leading cause of cancer-related death in women. Targeted therapy is a powerful strategy for treating hormone receptors (estrogen and progesterone) or human epidermal growth factor receptor 2 (HER2)-positive breast cancer. However, due to the lack of HER2 and hormone receptors in triple-negative breast cancer (TNBC), there is no effective targeted and precise treatment for TNBC except surgery and chemotherapy. Compared with other subtypes, TNBC is more aggressive and has potential for distant metastasis and is prone to relapse within 2 years after surgery [1]. The overall median survival of TNBC patients with recurrence and metastasis is only 13–18 months [2]. Therefore, the development of drugs and strategies for the treatment of TNBC is urgent.

Currently, some breakthroughs in cell death in malignant tumors have been proposed, such as ferroptosis [3, 4], which is an iron-dependent cell death driven by lipid peroxidation [5]. Several studies suggest that ferroptosis may be a native tumor-suppressive mechanism contributing to the antitumor function of p53, BAP1, and fumarase, which were all clinically relevant tumor suppressors [3, 6–8]. Strikingly, TNBC cells were susceptible to ferroptosis triggered by both cysteine deprivation and inhibition of glutathione peroxidase-4 (GPX4), a lipid peroxide-clearance enzyme [4]. Thus, induction of ferroptosis in TNBC could be a practical therapeutic approach in the future.

Hinokitiol, also known as β-Thujaplicin, is a natural tropolone derivative isolated from *Thuja Plicata* and *Chamaecyparis obtusa.* Hinokitiol can bind iron via its hydroxy ketone moiety as an iron chelator [9]. A variety of biological properties have been revealed, including anti-inflammatory [10, 11], antimicrobial [12–14], and anticancer potential [15–19]. Interestingly, whether the properties result from iron chelation has not caught attention. Iron is a redox-active essential element in almost all life, being involved in oxygen transport and electron transport in the mitochondrial electron transport chain (ETC) and DNA and protein synthesis. The effects of hinokitiol as an iron chelator can explain the inhibition of cell proliferation in breast cancer cells [17] and migration of melanoma [16], induction of S-phase arrest in colon cancer cells [18], and TNBC cells [15] and human hepatocellular carcinoma [19]-derived tumor growth. The downstream consequence was converged to be autophagic cell death and/or apoptosis [18, 19] or cell cycle arrest [15–17].

In our study, we surprisingly observed that the effects of hinokitiol against TNBC cell growth could be reversed by other iron chelators, deferoxamine (DFO) and deferiprone (DFP), but aggravated by iron addition in a concentration-dependent manner. The in vitro and in vivo results demonstrated that the formed hinokitiol-iron complex Fe(hino)_3_ boosted cellular lipid ROS levels and elicited ferroptosis. This study has demonstrated that Fe(hino)_3_ is a novel ferroptosis inducer, suggesting its promising therapeutic potential against TNBC and, very likely, other types of cancer.

## Results

### Hinokitiol, an iron chelator, -initiated cytotoxicity is reversed by two typical iron chelators, DFP and DFO, in TNBC cells

**Fig. 1:**
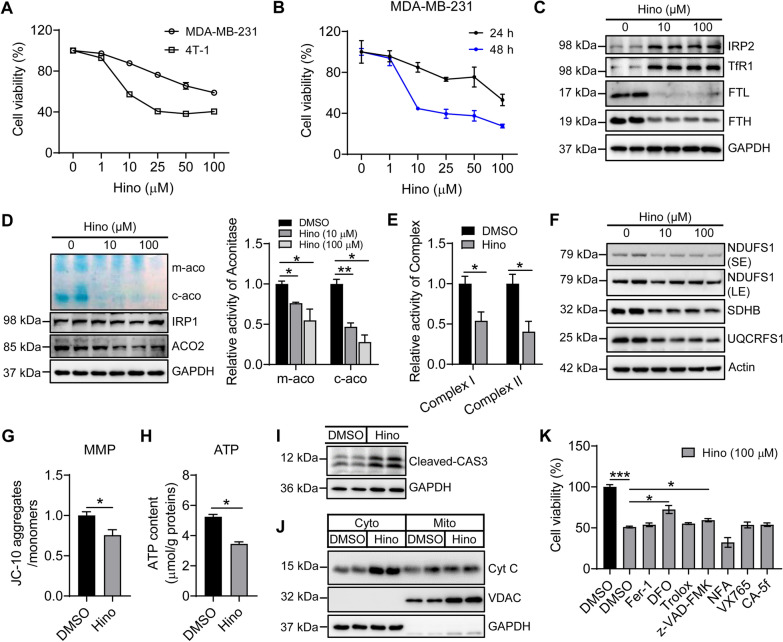
Iron chelator deferoxamine (DFO) inhibits hinokitiol (Hino)-induced apoptosis in tri-negative breast cancer cells (TNBCs). Two TNBCs cells lines, MDA-MB-231 and 4T-1, were used in (**A**), one MDA-MB-231 in (B-K). **A** Hino effect on cell viability, detected by CCK8, in a concentration-dependent manner 24 h post-Hino treatment (8,000 cells per well). **B** Hino effect on cell viability within 24 and 48 h post-Hino treatment (8,000 cells per well). **C** The expression of iron-related proteins IRP2, TfR1, FTL, and FTH in cells treated with Hino for 24 h. **D** The aconitase activities of cells treated with Hino for 24 h. m-aco, mitochondrial aconitase, c-aco, cytosolic aconitase. **E** The activities of mitochondrial electron transport chain (ETC) complexes (activities: complex I and II) in cells treated with 100 µM Hino for 24 h. **F** The protein expression of mitochondrial electron transport chain (ETC) complexes (NDUFS1, SDHB, and UQCRFS1, subunits of complex I/II/III, respectively) in cells treated with Hino for 24 h. **G**, **H** Mitochondrial membrane potential detected using JC-10, and ATP content in cells treated with 100 µM Hino for 24 h. **I**, **J** The levels of apoptosis-related proteins, cleaved caspase 3, and released cytochrome C from mitochondria to cytosol, detected by immunoblotting in cells treated with 100 µM Hino for 24 h. **K** DFO inhibited the Hino effect on cell viability. DMSO: dimethylsulphoxide as a vehicle; Fer-1 (1 µM): ferroptosis inhibitor ferrostatin-1; Trolox (100 µM): vitamin E derivative as an antioxidant; DFO (20 µM): iron chelator deferoxamine; z-VAD-FMK (25 µM): apoptosis inhibitor; NFA (2 µM): necrosis inhibitor necrosulfonamide; VX765 (10 µM): caspase-1 inhibitor belnacasan; CA-5f (2 µM): autophagy inhibitor. *, *p* < 0.05; **, *p* < 0.01, ***, *p* < 0.001

Given that iron is essential for cell growth and hinokitiol can bind iron efficiently, we examined the effect of hinokitiol on iron metabolism and cell growth. Human and mouse TNBC cells MDA-MB-231 and 4T-1 were treated with hinokitiol, respectively. Consistence with previous reports [20], hinokitiol effectively decreased the cell viability in a dose-dependent manner (Fig. 1A and B). The protein levels of iron regulatory protein 2 (IRP2), an iron sensor, and transferrin receptor (TfR1, for iron uptake), regulated post-transcriptionally by IRP2, were increased, while iron storage protein ferritin L and H subunits (FTL and FTH) were decreased, confirming an iron chelation action of hinokitiol and cellular iron-starvation response (Fig. 1C). Labile iron is critical for the iron-sulfur cluster (Fe-S) synthesis, which is lessened under iron-depleted conditions. The in-gel activity assays demonstrated that the activities of Fe-S containing proteins, mitochondrial (m-aco) and cytosolic (c-aco) aconitases, were decreased after hinokitiol treatment without changes in the protein levels (Fig. 1D). Consistent with these results, hinokitiol significantly reduced the Fe-S-dependent enzymatic activities of complex I and II of electron transport chain (ETC), and the Fe-S-containing subunits of complex I/II/III, NDUFS1, SDHB, and UQCRFS1, are all reduced, indicating the compromised mitochondrial function (Fig. 1E, F and Additional file 1: Fig. S1). Accordingly, the mitochondrial membrane potential (MMP) and ATP production, two critical indicators of mitochondrial integrity and bioenergetic function, significantly decreased (Fig. 1G and H). Further, we found that hinokitiol treatment induced apoptosis of TNBC cells, supported by the increased cleavage of caspase-3 and the release of cytochrome C from mitochondria to cytosol (Fig. 1I and J), which were in agreement with the previous studies [18, 19]. The results have firmly demonstrated the iron-chelation property of hinokitiol.

However, when we used a variety of cell death inhibitors to treat the cells following hinokitiol addition, we surprisingly found that the most effective inhibition of cell death resulted from treatment with iron chelators, DFO (Fig. 1K) and DFP (Additional file 1: Fig. S2), then apoptosis inhibitor z-VAD-FMK. However, the rescue effects of z-VAD-FMK did not change the iron status (Additional file 1: Fig. S3A–C), further supporting the hinokitiol function as an iron chelator. These data together suggest that hinokitiol-promoted cell death, at least, derived from not only apoptosis but also from another iron-dependent cell death in TNBC cells.

### Hinokitiol-iron complex fe(hino)_3_ induces cell death in TNBC cell lines at low concentrations

**Fig. 2:**
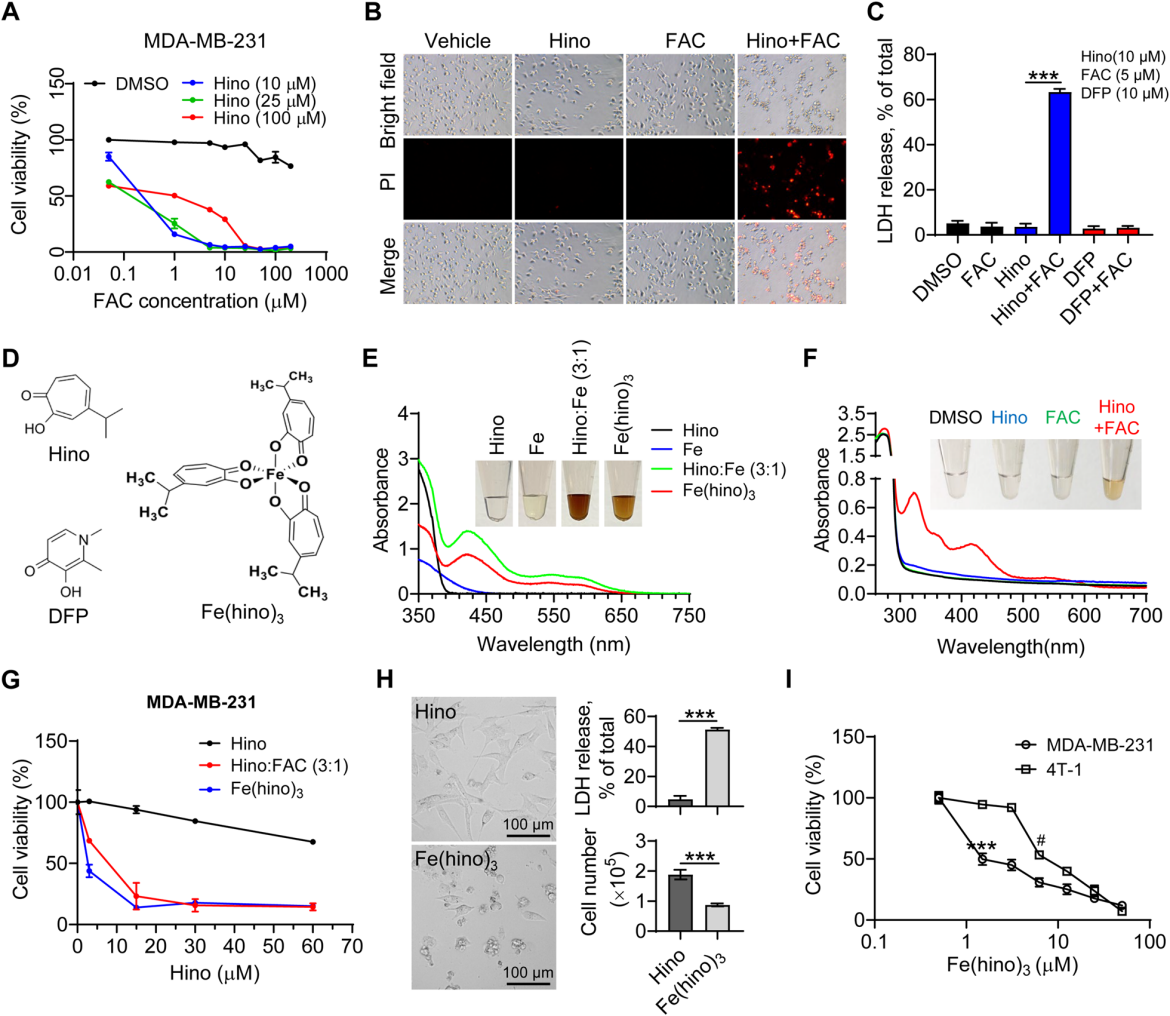
Hino/iron complex Fe(hino)_3_ induces cell death in TNBCs. **A** Cell viability in MDA-MB-231 treated with different concentrations of Hino and FAC for 24 h. **B**, **C** Propidium iodide (PI) staining (**B**) and lactate dehydrogenase (LDH) release (**C**) in MDA-MB-231 treated with Hino (10 µM), FAC (5 µM), or/and deferiprone (DFP as an iron chelator) (10 µM) for 24 h. **D** The structure of Hino, DFP, and Hino/iron complex Fe(hino)_3_ [9]. **E** Colors and UV/vis absorption spectra (260–700 nm range) of Hino, FeCl_3_ (1 mM), a mixture of Hino (3 mM) and FeCl_3_ (1 mM), and Fe(hino)_3_ (1 mM). **F** Colors and UV/vis absorption spectra (260–700 nm range) of the cell lysate of MDA-MB-231 treated with Hino (100 µM), FAC (33 µM), and Hino (100 µM) + FAC (33 µM) for 12 h. **G** Cell viability of MDA-MB-231 treated with different concentrations of Hino, Hino:FAC (3:1), or Fe(hino)_3_ for 24 h. The number of seeded cells is 10,000 per well. **H** Cell morphology, LDH release, cell number of MDA-MB-231 cells treated with Hino (15 µM) or Fe(hino)_3_ (5 µM) for 24 h. **I** Cell viability of MDA-MB-231 or 4T-1 cells treated with different concentrations of Fe(hino)_3_ for 24 h. *: compared to the untreated MDA-MB-231 cells; #: compared to the untreated 4T-1 cells. ***, *p* < 0.001; #, *p* < 0.05

To determine why iron-chelators DFP and DFO inhibited the effects of the iron-chelation property of hinokitiol, MDA-MB-231 cells were treated with different iron concentrations and hinokitiol. Interestingly, compared to hinokitiol or iron alone, the hinokitiol and iron co-treatment induced a dramatic decrease in cell viability (Fig. 2A). To further examine if the co-treatment caused more cell death, propidium iodide (PI), impermeable to live cells, was used to stain the dead cells. In accordance with the above observation, PI-staining showed that the co-treatment remarkedly increased PI fluorescence compared to the controls, indicating more membrane disruption by the co-treatment of hinokitiol and iron (Fig. 2B). Considering that the breakage of plasma membrane integrity induces the release of cytosolic components, we measured the released lactate dehydrogenase (LDH) as an indicator of cytotoxicity. Indeed, hinokitiol and iron co-treatment markedly increased the release of LDH into the cell culture medium. However, DFP, sharing a similar structure to hinokitiol as an iron chelator, did not induce LDH release when co-treated with iron (Fig. 2C), suggesting that the effects of cotreatment result from the toxicity of hinokitiol and iron together as a whole.

Hinokitiol contains a β-diketone moiety in the structure and forms complexes with various metal ions. This natural product rapidly binds iron to form a complex Fe(hino)_3_, as evidenced by an immediate change in color and UV-Vis spectra (from colorless to dark brown) at a 3:1 ratio (ref [9] and Fig. 2D and E). To examine whether the added iron and hinokitiol could form Fe(hino)_3_ within cells, the total protein extracts 12 h post-treatment were scanned by UV-Vis spectroscopy and presented an absorption peak at 425 nm (Fig. 2F), which is the characteristic absorption peak of Fe(hino)_3_, proving the presence of Fe(hino)_3_ within cells.

To further determine that the effects of hinokitiol and iron cotreatment resulted from the complex Fe(hino)_3_, we used the same concentration of these compounds to treat MDA-MB-231 cells. Surprisingly, both the complex Fe(hino)_3_ and co-treatment with hinokitiol and iron significantly and dramatically boosted the cytotoxicity compared with hinokitiol alone (Fig. 2G). LDH release data also verified the detrimental activity of Fe(hino)_3_ (Fig. 2C, H, and Additional file 2: Video S1), indicating that the plasma membrane was disrupted after Fe(hino)_3_ treatment. MDA-MB-231 cells were much more sensitive (> 100 fold) to Fe(hino)_3_ than to hinokitiol alone in a concentration-dependent manner (Fig. 2A and G). Relatively, the human TNBC cells MDA-MB-231 cells were even more susceptible than the murine TNBC 4T-1 cells (Fig. 2I). This deleterious effect was also verified in a few other tumor cells (Additional file 1: Fig. S4A and B). Thus, Fe(hino)_3_ exhibits highly cellular toxicity in TNBC cell lines.

### Fe(hino)_3_ triggers ferroptosis in TNBC cells

**Fig. 3:**
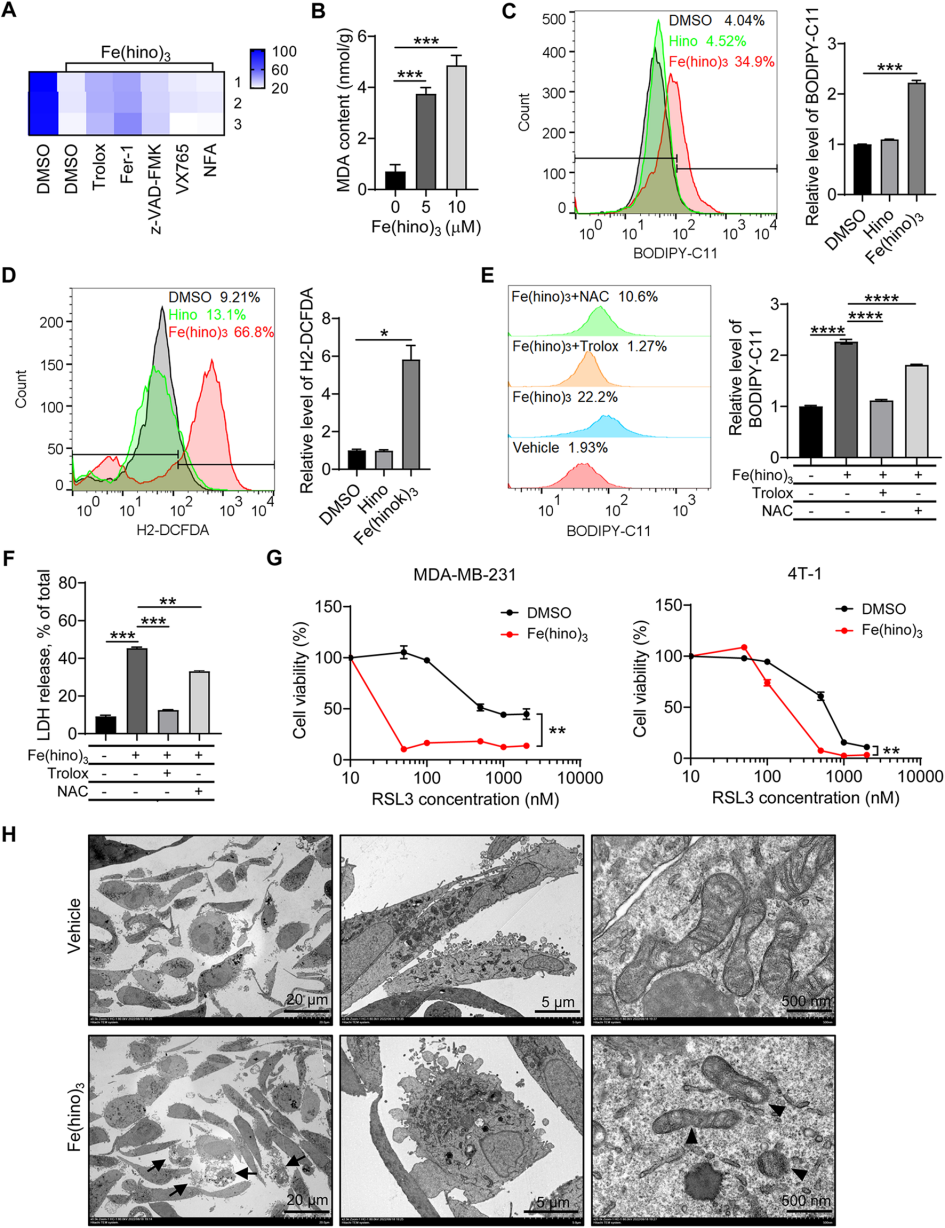
Fe(hino)_3_ triggers the ferroptosis of TNBCs. **A** Cell viability of MDA-MB-231 cells treated with 5 µM Fe(hino)_3_ following pretreatment with different inhibitors of cell death for 24 h. Trolox (200 µM), Fer-1 (5 µM), z-VDA-FMK (25 µM), VX765 (10 µM), NFA (2 µM). **B** Malondialdehyde (MDA) content in MDA-MB-231 cells treated with Fe(hino)_3_ for 24 h, measured with MDA detection kit. **C–D** Lipid ROS (**C**) and general ROS levels (**D**) detected by flow cytometry with BODIPY-C11 and H_2_-DCFDA, respectively, in MDA-MB-231 cells following treatment with Hino (15 µM) or Fe(hino)_3_ (5 µM) for 24 h. Right panel: Quantification of the fluorescence. **E**–**F** Lipid ROS levels and LDH release in MDA-MB-231 cells after Fe(hino)_3_ (5 µM) treatment for 24 h following 2-h pretreatment with ROS scavengers, NAC (5 mM), or Trolox (200 µM). **G** Cell viability of MDA-MB-231 or 4T-1 cells after co-treatment with Fe(hino)_3_ (5 µM) and different concentrations of RSL3 for 24 h. **H** Representative electron micrographs of MDA-MB-231 cells treated with vehicle or Fe(hino)_3_ (5 µM) for 24 h. Arrows indicate the dead cells and the ruptured cell membrane, and triangles indicate the damaged mitochondria. *, *p* < 0.05; **, *p* < 0.01; ***, *p* < 0.001; ****, *p* < 0.0001

To determine the mode of cell death induced by Fe(hino)_3_ in TNBC cells, we used different cell death inhibitors to intervene in the cell death process. The results showed that ferroptosis inhibitors ferrostatin-1 (Fer-1) and Trolox significantly inhibited Fe(hino)_3_-induced cell death (Fig. 3A), suggesting that Fe(hino)_3_ triggered ferroptosis in TNBC cells. Malondialdehyde (MDA) is a major degradation product of lipid hydroperoxides as an indicator of lipid peroxidation. The cellular content of MDA was significantly increased after Fe(hino)_3_ treatment (Fig. 3B). To directly determine the levels of cellular reactive oxygen species (ROS), we used two fluorescent probes, BODIPY-C11 and H_2_-DCFDA, as indicators of lipid peroxides and general ROS, respectively. Both levels were significantly increased when MDA-MB-231 cells were treated with Fe(hino)_3_, but no significant changes with hinokitiol alone (Fig. 3C and D). Hydrophilic N-acetyl-L-cysteine (NAC) and hydrophobic Trolox are two effective ROS scavengers. Both significantly attenuated the Fe(hino)_3_-induced ROS production and abolished LDH release (Fig. 3E and F). Of both, the latter one, Trolox, exhibited greater inhibition, suggesting that lipid peroxidation played a more critical role in Fe(hino)_3_-induced cell death, which was further verified in other carcinoma cell lines (Additional file 1: Fig. S4C–E). RSL3 is a ferroptosis inducer and can inhibit the activity of GPX4. We found that RSL3 treatment drastically increased the sensitivity of the two lines of TNBC cells to Fe(hino)_3_ treatment (Fig. 3G). Morphologically, the membrane integrity was examined by transmission electron microscopy. The results showed normal nuclei, shrinking mitochondria with increased membrane density, and cytoplasmic membrane rupture (Fig. 3H). Taken together, we demonstrated that Fe(hino)_3_ induces a ferroptotic phenotype in TNBC cells.

### Fe(hino)_3_ is a redox-active complex to inducelipid peroxidation through the Fenton reaction and depletes glutathione (GSH)

**Fig. 4:**
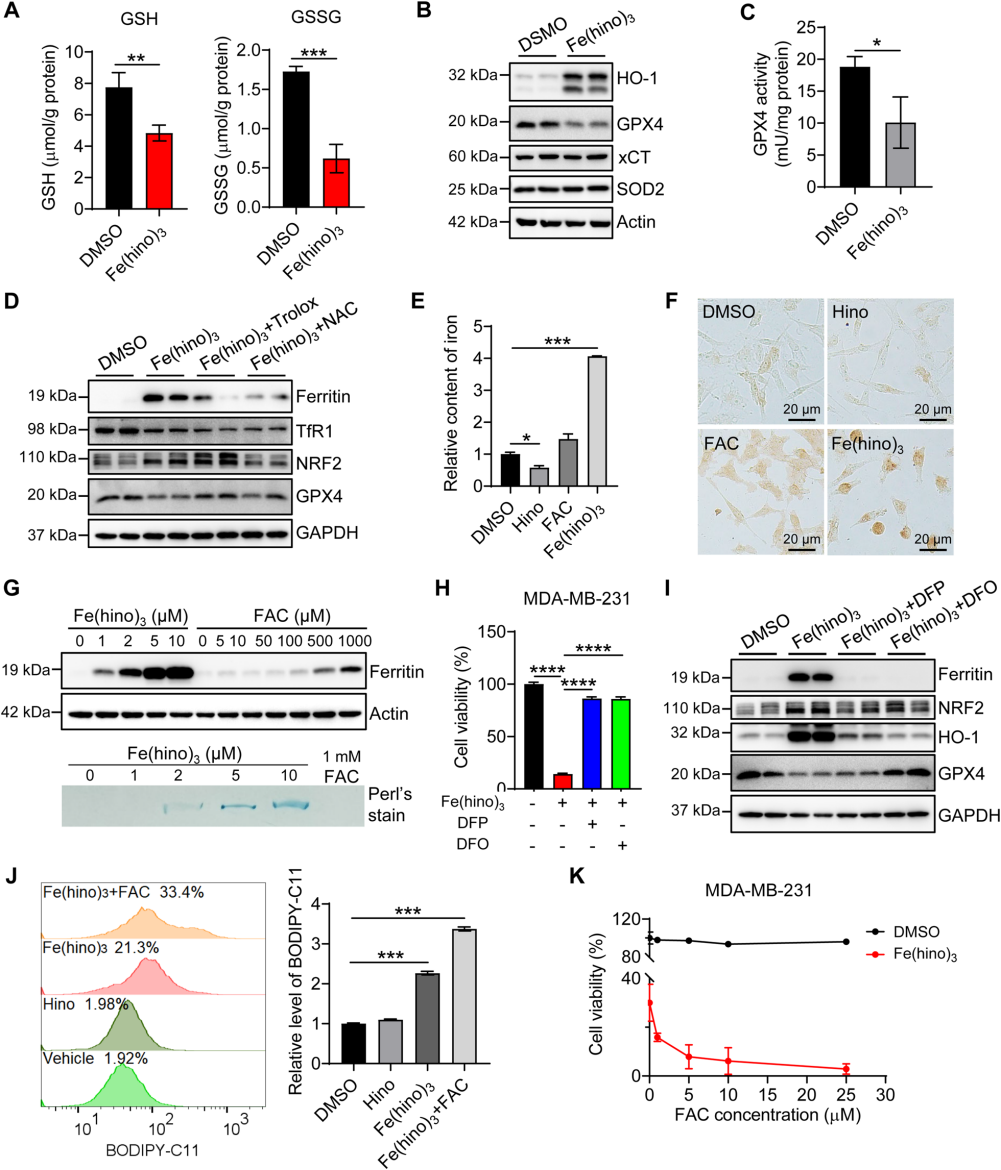
Fe(hino)_3_ -induced ferroptosis is iron-dependent. **A**–**C** The content of GSSG and GSH (**A**), the expression of HO-1, GPX4, xCT, and SOD2 (**B**), and the enzyme activity of GPX4 (**C**) in MDA-MB-231 cells treated with Fe(hino)_3_ (5 µM) for 24 h. **D** The expression of Ferritin, TfR1, NRF2, and GPX4 in MDA-MB-231 cells treated with 5 µM Fe(hino)_3_ alone or with Trolox (200 µM) or NAC (5 mM) together for 24 h. **E** Total iron content measured by ferrozine assays in MDA-MB-231 cells treated with Hino (15 µM), ferric ammonia citrate (FAC) (5 µM), and Fe(hino)_3_ (5 µM) for 24 h. **F** Cellular nonheme iron levels were detected using DAB-enhanced Perl’s staining. **G** Upper panel: Ferritin (FTH subunit) expression in MDA-MB-231 cells treated with different concentrations of FAC or Fe(hino)_3_ for 24 h, detected by immunoblotting. Lower panel: the iron level in ferritin was detected by Perl’s stain. **H**–**I** Cell viability (H) and protein expression (**I**) of MDA-MB-231 cells after 24-h incubation with Fe(hino)_3_ (5 µM) or/and DFO (50 µM) or DFP (50 µM). **J** The lipid ROS in MDA-MB-231 cells treated with Fe(hino)_3_ (2 µM) alone or with FAC (5 µM) together for 24 h, detected by flow cytometry and indicated by BODIPY-C11. **K** Cell viability after cotreatment with Fe(hino)_3_ (2 µM) and FAC for 24 h. *, *p* < 0.05; **, *p* < 0.01; ***, *p* < 0.001; ****, *p* < 0.0001

The GSH-GPX4 axis is a crucial pathway for scavenging lipid peroxides to inhibit the occurrence of ferroptosis. To examine the redox activity of Fe(hino)_3_ on physiologic substrates, levels of the well-described indicator of oxidative stress, GSH, were determined. After 24 h incubation with MDA-MB-231 cells, Fe(hino)_3_ reduced the contents of both GSH and GSSG/GSH and the protein level and activity of GPX4 (Fig. 4A–C), but had no effect on the protein level of cystine transporter xCT/SLC7A11 (Fig. 4B). Meanwhile, antioxidant Trolox and NAC rescued the cell viability (Fig. 3F) and restored the production of GPX4 with higher efficiency post-Trolox than post-NAC treatment (Fig. 4D). In accord with this, NRF2, a master transcriptional factor to respond to oxidative stress, was activated following Fe(hino)_3_ treatment and reversed after NAC co-treatment, but not Trolox co-treatment. The activity of increased NRF2 was viewed by increased heme oxygenase 1 (HO-1) (Fig. 4B), which is one of the members of the NRF2 regulon. Thus, Fe(hino)_3_ is supposed to be a redox-active complex to induce lipid membrane peroxidation.

To explore whether the redox-active property of Fe(hino)_3_ resulted from the chelated iron, we first detected cellular iron content and intracellular iron metabolism. Astonishingly, the same concentration of iron (5 µM) as in ferric ammonia citrate (FAC) and in Fe(hino)_3_ provoked significantly different iron content in the cytosol, 4 folds more when treated with Fe(hino)_3_ than with FAC (Fig. 4E). The result was further confirmed by direct 3,3’-diaminobenzidine (DAB)-enhanced Perl’s staining (Fig. 4F), suggesting that the lipophilic feature of Fe(hino)_3_ accelerates the hinokitiol-chelated iron to enter cells. More interestingly, compared to the FAC, Fe(hino)_3_ treatment dramatically increased the protein level of ferritin with greater than 500 folds effectivity, thus, ferritin was loaded with a massive amount of iron (Fig. 4G). The iron chelator DFO or DFP significantly reversed the detrimental effects induced by Fe(hino)_3_ (Fig. 4H and Additional file 1: Fig. S4F). Additionally, Fe(hino)_3_ presented in the cell pellets from Fe(hino)_3_-treated cells, not from the cells co-treated with Fe(hino)_3_ and DFO or DFP (Additional file 1: Fig. S5A), indicating that DFO or DFP chelates iron released from Fe(hino)_3_. The UVI spectrum analysis also showed that DFO or DFP treatment abolished the characteristic peak at 425 nm of Fe(hino)_3_ in cell culture medium with higher efficiency by DFO than by DFP (Additional file 1: Fig. S5B). Thereby DFP and DFO rescue the cells from Fe(hino)_3_ treatment by chelating the active iron released by Fe(hino)_3_ and iron release from Fe(hino)_3_ is dynamic. And more, both DFP and DFO addition restored the protein level of GPX4 and decreased protein levels of ferritin, NRF2, and HO-1 (Fig. 4I). The highly oxidized cellular status post-Fe(hino)_3_ treatment was also revealed by fluorescent probe BODIPY-C11 (Fig. 4J), indicating the massive lipid peroxidation through Fe(hino)_3_. Consequently, the cell viability was dramatically reduced by additional iron addition following Fe(hino)_3_ treatment (Fig. 4K).

Together, these data provide solid evidence that Fe(hino)_3_ is a redox-active complex to induce lipid peroxidation through the Fenton reaction and disrupts the redox homeostasis, which toxicity is iron-dependent.

### Fe(hino)_3_ exerts ferroptosis-mediated antitumor efficacy in vivo

**Fig. 5:**
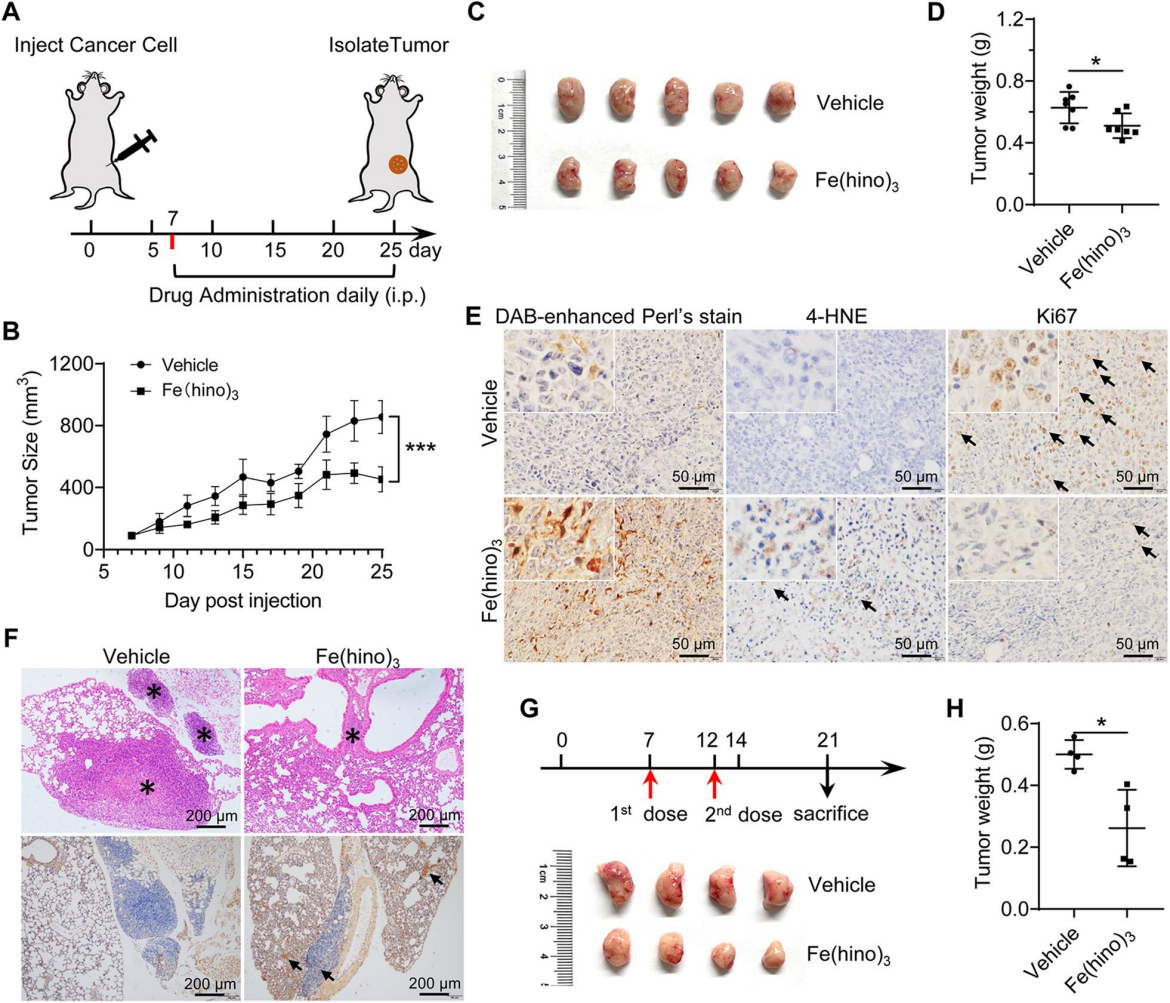
Effects of Fe(hino)_3_ on TNBCs-induced tumor growth. **A** 4T-1 cells were orthotopically injected into the mammary pad of female Balb/c mice. A schematic graph to use Fe(hino)_3_ (2 mg/kg, i.p.) in a 4T-1 cell orthotopic tumor model. **B** Tumor size was recorded on the indicated days. **C** Representative isolated-tumor images from each group on day 25 after treatment with Fe(hino)_3_ for 18 days. **D** The weight of isolated tumors on day 25. **E** The content of 4-HNE and expression of Ki67, detected by immunohistochemistry, and the iron level, detected using DAB-enhanced Perl’s staining in tumors. **F** Upper panel: the tumors metastasized to the lungs, revealed by H&E staining and marked with *. Lower panel: the DAB-enhanced Perl’s iron staining and the iron enrichment marked with arrows. **G**, **H** Representative isolated-tumors and tumor weight from each group on day 21 after two shots with Fe(hino)_3_ through intratumor injection. *, *p* < 0.05; ***, *p* < 0.001

To further test the therapeutic potential of Fe(hino)_3_in vivo, mouse TNBC 4T-1 cells were injected into BALB/c mouse mammary fat pad. When the tumor reached 80–100 mm^3^ in size (~ 7 d post-injection), the mice were randomly divided into two experimental groups: the vehicle and Fe(hino)_3_ (i.p. 2 mg/kg) groups (Fig. 5A). After an additional 18 d, we observed a significantly smaller tumor size and lighter weight in the Fe(hino)_3_ group than in the vehicle group (Fig. 5B–D). To determine whether the anti-tumor efficacy resulted from Fe(hino)_3_-induced ferroptosis, tumor iron was detected using DAB-enhanced Perl’s staining. More iron was enriched in tumors in the Fe(hino)_3_ group than in the vehicle group (Fig. 5E). And 4-Hydroxynonenal (4-HNE), an α, β-unsaturated hydroxyalkenal that is produced by lipid peroxidation, was significantly increased post-Fe(hino)_3_ administration. This result was further verified by the expression of Ki67, an indicator of cell proliferation (Fig. 5E and Additional file 1: Fig. S6A–C). In addition, Fe(hino)_3_ also suppressed breast cancer-derived lung metastasis, and the iron-staining showed that more iron was enriched in the lung in the Fe(hino)_3_ group (Fig. 5F and Additional file 1: Fig. S6D), suggesting the Fe(hino)_3_-based blockage of the lung metastasis of breast tumor. Lastly, we targeted the tumors with Fe(hino)_3_ two shots within 5 days following the tumor formation. After additional 9 days, the tumors were harvested, and their size and weight were measured (Fig. 5G, upper panel). The results showed a more significant reduction of the breast tumors compared to the effect of intraperitoneal administration (Fig. 5G and H). Thus, Fe(hino)_3_ exerts anti-TNBC and anti-metastasis efficacy *in vivo.*

To examine the safety and tolerance of Fe(hino)_3_ usage, i.e., whether Fe(hino)_3_ is toxic to normal non-tumor-bearing mice or other tissues in tumor-bearing mice following Fe(hino)_3_ administration, we tested some physiological parameters, including body weight and the sizes and morphology of some organs. Any abnormalities were not observed, such as the body weight (Additional file 1: Fig. S7A) and the size or length of the small intestine and spleen (Additional file 1: Fig. S7B, C). In the tumor-bearing mice, the serum biochemical indicators, including aspartate aminotransferase (AST), alanine aminotransferase (ALT), creatine kinase MB (CK-MB), glucose (GLU), and urea also maintained unchanged except the slightly but significantly low levels of creatinine after Fe(hino)_3_ treatment (Additional file 1: Fig. S7D). The body weight showed mild change (Additional file 1: Fig. S7E), suggesting that the mice were tolerant to Fe(hino)_3_ treatment with the given dosage.

## Discussion

**Fig. 6:**
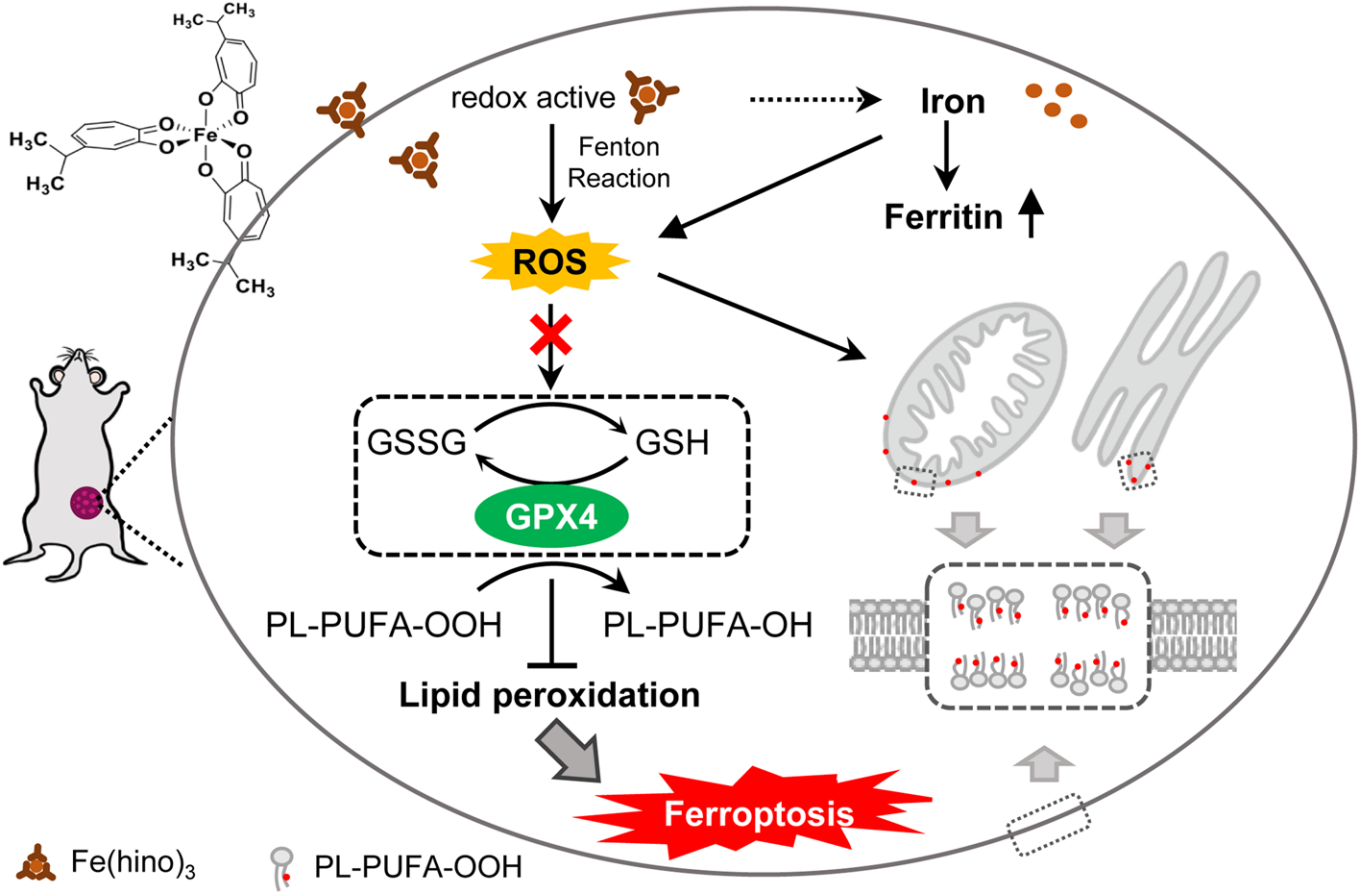
A schematic diagram that presents Fe(hino)_3_ -induced ferroptosis in tumors. Hino acting as an iron chelator forms a redox-active complex with iron. Upon the complex entering the cell, Fe(hino)_3_ (major) and released iron (minor) promote the generation of hydroxyl radicals through the Fenton reaction, resulting in the occurrence of lipid peroxidation and reduced GSH-GPX4 production, ultimately leading to ferroptosis in TNBCs

Hinokitiol, a small-molecule natural product, is considered an iron chelator with anti-inflammatory [21], antioxidant [22], antibacterial [23, 24], and anti-fungal [25] properties. In this study, we found that hinokitiol acting as an iron chelator, formed a redox-active complex with iron. This complex Fe(hino)_3_ functions as a ferroptosis inducer to inhibit TNBC growth, summarized in Fig. 6. The inhibition effect is based on lipid peroxidation through the Fenton reaction and reducing GSH-GPX4 production and can be reversed by other redox-inactive iron chelators. This novel property presents intriguing application potential in the treatment of TNBC.

Up to date, no available in vivo data show the anti-TNBC effects of hinokitiol, likely, due to the resistance of TNBC cells to hinokitiol, including SUM159 and MDA-MB-231 cell lines [15]. The in vivo anti-tumor effect of hinokitiol was observed when hinokitiol was injected within 1–3 days following inoculation of other tumor cell types [16, 18]. The mechanism was proposed by cell cycle arrest [15] or apoptosis/autophagy [18, 19]. Unexpectedly, we found that hinokitiol-iron complex Fe(hino)_3_ induced ferroptosis by boosting the iron-dependent lipid peroxidation in TNBC in vitro and in vivo models. Notably, Fe(hino)_3_ was administrated 7 days after inoculation of the TNBC cells when the tumor was formed up to 100 mm^3^. The effects of more and higher dosages will be further investigated.

Hinokitiol was found to chelate iron or other metals via its hydroxy ketone moiety, which exerts various anti-microbial activities decades ago [13, 26]. Recently, Grillo et al. have demonstrated that hinokitiol binds iron (ratio 3:1) to form a natural Fe(hino)_3_ complex and partially mimics the function of missing membrane transporters of iron to mediate site- and direction-selective restoration of iron transport [9, 27]. Yeast was alive when treated with iron (FeCl_3_, 10 µM) alone but died when cotreated with iron (10 µM) and hinokitiol (32 µM) [9]. The cytotoxic effect of hinokitiol might be explained by its prooxidant properties: the hinokitiol/transition metal complex generates ROS [28]. In our study, co-treatment of hinokitiol with iron dramatically increased the sensitivity of TNBC cells compared to hinokitiol-only treatment. This effect was further boosted with the addition of more iron. We propose that hinokitiol and iron form a redox-active complex, like Di-2-pyridylketone 4,4-dimethyl-3-thiosemicarbazone (Dp44mT) and iron, to induce free radicals under redox condition [29]. Interestingly, when more hinokitiol was present, the lethal effect of Fe(hino)_3_ was suppressed. The action of hinokitiol in the biological system seems biphasic, as suggested by Qin and his colleagues [30]. On the one hand, it forms the lipophilic and redox-active iron complexes, which quickly enter cells and facilitate •OH production through the Fenton reaction; on the other hand, when the ratio of hinokitiol to iron is higher than 3, redox-inactive iron complexes are proposed [30]. Our data support this biphasic hypothesis. Hinokitiol has been shown to act as an antioxidant by scavenging free radicals [31], except its metal chelation property as discussed above. The bifunctional characteristics of hinokitiol are intriguing and explain that the high concentration of hinokitiol makes Fe(hino)_3_ less toxic in our study.

Fe(hino)_3_ is characterized by high lipophilicity and non-polarity [32]. Upon entering cells, Fe(hino)_3_ might rapidly increase cellular redox iron, which is highly deleterious due to the hydroxyl free radicals to induce lipid peroxidation. Though Fe(hino)_3_ may release iron, evidenced by increased ferritin, a large portion of Fe(hino)_3_ functions as a whole, supported by the cellular characteristic spectrometry absorption of Fe(hino)_3_. The cellular condition, very likely, provides a perfect redox environment for Fe(hino)_3_ to actively generate free radicals along the membrane system, thus inducing ferroptosis. Particularly, iron addition following Fe(hino)_3_ treatment further enhanced the tumor-killing activity, suggesting the active dynamics of Fe(hino)_3_, which means that hinokitiol easily captures iron once the iron is released from the complex Fe(hino)_3_. In addition, once released, iron as a prosthetic group of arachidonic acid lipoxygenases (ALOX) might enhance ALOX to trigger lipid peroxidation and, potentially, ferroptosis [33, 34]. Unexpectedly, this toxicity is favored in tumor, proved by correlation of the enrichment of iron and less proliferation of tumor cells. And the serum biochemical parameters were normal in the Fe(hino)_3_-treated tumor-bearing mice. Therefore, Fe(hino)_3_ is a promising potential for an efficient TNBC therapy.

The key cellular redox systems, such as SLC7A11/GSH/GPX4, the NAD(P)H/FSP1/ubiquinone, and the DHODH/CoQH_2_ axis, constantly surveil and neutralize oxidative damage in cellular membranes and mitochondria [35–37]. Emerging evidence shows the great potential of ferroptosis inducers for cancer therapy, particularly for eradicating aggressive malignancies that are resistant to traditional therapies [38–40]. TNBC is an aggressive and heterogeneous subset of breast cancer that is resistant to existing targeted therapies. It was previously reported that TNBC cells are particularly sensitive to GSH depletion [41]. In this study, we found that Fe(hino)_3_ not only induced GSH depletion, but also GSSG/GSH. Very likely, it resulted from the cell membrane disruption after Fe(hino)_3_ treatment, and GSH and GSSG are small molecules to be easily leaky out of the cells, consistent with the increased LDH release. GPX4 and ferroportin have been shown significantly lower expression in TNBC sections compared to other breast cancers [4], providing a therapeutic environment for ferroptosis. Peroxidation of n-3 and n-6 polyunsaturated fatty acids in the tumor environment enhances ferroptosis-mediated anticancer effects [42, 43]. Besides, the polyunsaturated fatty acids docosahexaenoic acid (DHA) and arachidonic acid (AA) were elevated in TNBC [44, 45], in agreement with their high susceptibility to ferroptosis. Taken together, Fe(hino)_3_, acting as a ferroptosis inducer, brings therapeutical potential against TNBC.

## Conclusions

In conclusion, our study found that Fe(hino)_3_ has the anti-TNBC ability via inducing ferroptosis. Our work suggests a potential candidate as an efficient therapy in the treatment of TNBC.

## Materials and methods

### Reagents

Hinokitiol was purchased from Tokyo Chemical Industry Co. (Tokyo, Japan). Ferrostatin-1 and RSL3 were purchased from Aladdin (Shanghai, China). NFA, VX765, and z-VAD-FMK were obtained from Selleck (Shanghai, China). The other used compounds included Trolox (Med Chem Express, Shanghai, China), CA-5f (Melliun, Dalian, China), DFO, and DFP (Sigma-Aldrich Inc, St Louis, MO).

### Cell lines and culture

Human breast cancer cell (MDA-MB-231, MCF-7), mouse mammary carcinoma cell (4T-1), human gastric carcinoma cells (MGC803, BGC823, and SGC7901), human myelogenous leukemia cell (K562) were purchased from the American Tissue Culture Collection (ATCC). These cell lines were cultured in high glucose DMEM supplemented with 10% FBS, penicillin (100 U/mL), and streptomycin (100 mg/mL) at 37 ℃ in, except for K562 which was cultured in medium RPMI 1640 (Solarbio Science & Technology Co. Ltd., Beijing, China) with 10% FBS. All cell lines were maintained at 37 °C in a 5% CO_2_ tissue culture incubator (Thermo Fisher Scientific., Waltham, MA).

### Synthesis of Fe(hino)_3_

Fe(hino)_3_ was synthesized according to a previous publication [9, 32]. In brief, iron (III) chloride (560.2 mg, 2.08 mmol) and hinokitiol (1.026 g, 6.24 mmol) were mixed in 10 mL ethanol. It was vigorously stirred for 2 hs at room temperature to give a cherry red purple-colored suspension. The product was collected via filtration and washed with ethanol, and then dried in a fume cupboard to yield the product as a purple solid Fe(hino)_3_. Characterization was carried out by spectrometry.

### In vivo tumor formation

To generate the orthotopic tumor xenograft model, 1 × 10^4^ 4T-1 cells were suspended in 50 µL of DMEM and orthotopically injected into the mammary fad of 6 weeks old female Balb/c mice. Seven days after the inoculation, the mice were divided into two groups for the treatments of the vehicle or Fe(hino)_3_ (2 mg/kg), which were administered via intraperitoneal injections every day for 18 days. For targeted treatment, an equivalent of 40 µg Fe(hino)_3_ per dose in a total 50 µL volume was injected directly into tumors. The second shot was given after 5 days. At the end of the experiment, the mice were sacrificed, and the tumors were measured. The tumor volume (V) was calculated as follows: V = [(length) × (width) × (width)]/2. The Fe(hino)_3_ was first prepared as a stock solution (50 mM, in DMSO). This solution was diluted with an aqueous solution containing 5% DMSO and 2% Tween-80. The animal experiments were approved by the Animal Research Committee, Nanjing University (Jiangsu, China). All the experimental procedures were carried out according to the guidelines of the National Institutes of Health Guide for the Care and Use of Laboratory Animals.

### Western blot analysis

The harvested cells were lysed in 1% NP-40, 125 mM Tris, pH 6.8, containing a protease inhibitor cocktail (Roche, Basel, Switzerland). For western blotting, 25–50 µg total protein was loaded into 10–12% SDS-PAGE per lane and analyzed by immunoblotting as described previously [46]. The primary antibodies included: TfR1 antibody (cat#136,800) from Zymed (San Francisco, CA), SDHB (cat#ab178423), cleaved-caspase3 (cat#ab179517) from Abcam (Cambridge, MA), ACO2 (cat#11134-1-AP), NDUFS1 (cat#12444-1-AP), UQCRFS1 (cat#1843-1-AP), NRF2 (cat#16396-1-AP), HO-1 (cat#66743-1-Ig) from Proteintech Group Inc. (Chicago, IN), anti-FTL, FTH, IRP1, and IRP2 (polyclonal, self-made, raised from rabbits) [47].

### Cell viability assays

According to the manufacturer’s instructions, a CCK-8 Cell Viability Assay Kit (Vazyme, Nanjing, China) was used for cell viability assays. The amount of formazan produced is directly proportional to the number of living cells and is measured by absorbance at 450 nm.

### Ferrozine iron assays and ferritin iron gel assays

Iron content was measured using a colorimetric ferrozine-based assay with some modifications [48, 49]. Briefly, 11 µL HCl (11.6 M) was added to 50 µL cell lysate (400 µg total protein). The mixed sample was heated at 95°C for 20 min, then centrifuged at 15,000 g for 10 min. The supernatant was transferred very gently into fresh tubes. Ascorbate was added to reduce the Fe (III) into Fe (II). After 2 min of incubation at room temperature, ferrozine and saturate ammonium acetate (NH_4_Ac) were sequentially added to each tube and the absorbance was measured at 562 nm (Potenov PT3502B, Beijing, China) within 30 min.

To detect ferric iron loading in ferritin, the protein was extracted from cells. The same amount of total protein (50 µg) was heated at 70℃ for 10 min, then centrifuged at 15, 000 g for 10 min. The supernatant was loaded on each lane and separated on a native-PAGE gel. The gel was stained with Perl’s staining solution (2% K_4_[Fe(CN)_6_] and 2% HCl) at room temperature overnight. The amount of iron loaded in ferritin was visible as blue bands.

### Histological staining

In H&E staining, tissue sections were dealt with the dewaxed and hydrated steps, followed by staining with hematoxylin for 5 min, then in Eosin Y for 5 min. In DAB-enhanced Perl’s staining, tissue sections were dewaxed and hydrated. The sections were then treated with freshly prepared acid ferrocyanide solution for 10–30 min, washed well in distilled water, and followed by DAB staining to enhance the color rendering. Tissue sections were stained with hematoxylin for an additional 10 min to stain the nuclei blue.

### Transmission electron microscopy

The cell samples were harvested at the indicated time. Firstly, the cell medium was replaced with warm 2.5% glutaraldehyde in 0.1 M Sodium Cacodylate buffer (pH 7.4) for 30 min at room temperature, then moved to 4℃ for 30 min. After this, the cells were washed with 0.1 M Na Cacodylate 3 times and were scraped to get the cell pellets. The fixed cells were pre-embedded with 1% Agarose. Next, the samples were fixed in 1% osmium tetroxide in 0.1 M Na Cacodylate for 1 h. Samples were then dehydrated through a graded series of ethanol from 50 to 100%, followed by replacing ethanol with propylene oxide. Keep samples in 50% propylene oxide and 50% Epon resin (1:1 mix) for 1 h and then in pure Epon. The next day, repeat the previous step once with a fresh Epon. Samples were transferred to fresh Epon in molds or Beem capsules (remove air bubbles) and were left in the oven for at least 24 h at 60°C. Samples were observed and captured in a HITACHI7800 transmission electron microscope at 80 kV.

### LDH release assays

Cell rupture was analyzed by detecting the activity of lactate dehydrogenase (LDH) released into the supernatant of the cell culture. LDH measurement was conducted with the LDH Cytotoxicity Assay Kit (Beyotime, Beijing, China) according to the manufacturer’s instructions.

### Flow cytometric analysis

The cells were seeded in 6-well plates at a density of 5 × 10^5^ per well and cultured overnight before treatment. Flow cytometry was performed on a FACS Calibur instrument according to the manufacturer’s protocol (BD Biosciences, NJ). Annexin V-fluorescein isothiocyanate (FITC) and propidium iodide (PI) staining were used to detect cell apoptosis, H_2_-DCFDA to measure intracellular ROS, and BODIPY-C11 to detect the level of lipid peroxidation. All data were analyzed using FlowJo software.

### Measurement of the enzymatic activities of aconitase and complex I/II

In-gel aconitase activity assays were performed as described previously [46]. The NADH dehydrogenase activity of isolated mitochondria was measured using a complex I enzyme activity microplate assay kit (ab109721, Abcam, Shanghai, China). The complex II assay was performed with the Succinate-coenzyme Q reductase activity assay kit (Comin Biotechnology Co., Suzhou, China) following the manufacturer’s instructions.

### Determination of mitochondrial membrane potential (MMP) level and ATP content

MMP levels were determined using the JC-10 mitochondrial membrane potential detection kit (Solarbio Science & Technology Co. Ltd., Beijing, China). The fluorescent intensity (excitation 490 nm, emission 520 nm) was determined using a fluorescence microplate reader. ATP levels were measured using an ATP content assay kit (Beyotime Biotech). The bioluminescence assay is based on the reaction of ATP with recombinant firefly luciferase and its substrate luciferin.

### Statistics analysis

Data were presented as mean ± SD, all the experiments were repeated more than three times independently. Student’s t-test or one-way analysis of variance (ANOVA) was done using GraphPad Prism 8. Significance was considered at *p* < 0.05.

## Supplementary Information

**Additional file 1: Figure S1. **Effect of hinokitiol (Hino) on the expression of iron-related genes in mouse tri-negative tumor cells 4T-1. Ndufs1, SdhB, and Uqcrfs1 are Fe-S proteins as one of the subunits of complex I/II/III, respectively. IscU is a scaffold protein for Fe-S biosynthesis. TfR1: transferrin receptor 1. 4T-1 cells were treated with Hino for 24 h. **Figure S2.** DFP inhibits the Hino effect on cell viability of MDA-MB-231. Cell viability of MDA-MB-231 treated with Hino (100 µM) alone or co-treated with Hino (100 µM) and DFP (50 µM) for 24 h. DFP: Deferiprone. *, *p *< 0.05. **Figure S3. **Hino functions as an iron chelator resulting in decreases of aconitase activity, protein expression of ETC complexes, and mitochondrial membrane potential in cells. (A) The expression of NDUFS1, SDHB, UQCRFS1 and activities of mitochondrial aconitase (m-aco) and cytosolic aconitase (c-aco) in MDA-MB-231 cells treated with Hino (100 µM) or/and z-VAD-FMK (50 µM) for 24 h. (B-C) The MMP levels and ATP levels in MDA-MB-231 cells treated with Hino (100 µM) or/and z-VAD-FMK (50 µM) for 24 h. *, *p*<0.05. **Figure S4. **Fe(hino)_3_ induces ferroptosis and addition of iron aggravates the effects in various tumor cells. (A) Cell viability of human gastric cancer cell lines (BGC823, SGC7901, MGC803) after cotreatment with Hino (10 µM) and different concentrations of FAC for 24 h. (B) Cell viability after treatment with Fe(hino)_3_ (3 µM) alone or plus FAC (6 µM) for 24 h in human gastric cancer cell lines, breast cancer cells (MCF-7), and myelogenous leukemia cells (K562). (C) LDH release of BGC823 cells after 24-h incubation with Fe(hino)_3_ (5 µM) or/and Trolox (200 µM). (D) The lipid ROS in BGC823 cells treated with Fe(hino)_3_ (5 µM) alone or withTrolox (200 µM) together for 24 h, detected by flow cytometry with BODIPY-C11.(E) The lipid ROS in K562 cells treated with Fe(hino)_3_ (5 µM) alone or with Trolox (200 µM) together for 24 h. (F) Cell viability and LDH release of BGC823 cells after 24-h incubation with Fe(hino)_3_ (5 µM) or/and DFO (50 µM) or DFP (50 µM). *, *p* < 0.05; **, *p* < 0.01; ***, *p* < 0.001. **Figure S5. **Fe(hino)_3_ releases iron that is efficiently chelated by DFP or DFO under cellularcondition. (A) The color of BGC823 cell pellets treated with Fe(hino)_3_ (10 µM) or/and DFO (50 µM) or DFP (50 µM) for 12 h. (B) Fe(hino)_3_ was diluted in the cell culture medium (DMEM+10% FBS) with a final concentration of 10 µM. DFO (final con. 50 µM) or DFP (final con. 50 µM) was added. After incubation at 37°C for 8 h, the samples were analyzed by UVI spectrum. **Figure S6.** The quantification for Figure 5E and F. The quantification of DAB-enhanced Perl’s stain positive area (A), of immunohistochemistry to present the percentage of 4-HNE positive area (B) and Ki-67 positive cells (C) in tumor tissue of Vehicle or Fe(hino)_3_ group in Figure 5E. (D) The quantification of the lung metastasis (tumor area/total lung tissue area) for Figure 5F. *, *p* < 0.05; **, *p* < 0.01; ****, *p* < 0.0001. **Figure S7.** Fe(hino)_3_ treatment shows no significant toxicity in mice. (A-C) Mice were intraperitoneally injected with Hino (5 mg/kg) or Fe(hino)_3_ (2 mg/kg). The same volume of an aqueous solution containing 5% DMSO and 2% Tween-80 as a vehicle indicated. (A) The body weight, (B) colon length, and (C) spleen volume of the mice. (D-E) Biochemical indicators after Fe(hino)_3_ (2 mg/kg) treatment. 4T-1 cells wereorthotopically injected into the mammary pad of female Balb/c mice. (D) No obvious side effects on the liver, kidney, and heart function in the Fe(hino)_3_ group except creatinine with mild and significant decrease. (E) The body weight of the mice harboring tumors, recorded every other day. *, *p* < 0.05.

**Additional file 2: Video S1. **The morphological changes of MDA-MB-231 cells after Fe(hino)_3_ treatment. The morphological changes of MDA-MB-231 cells after Fe(hino)_3_ (5 μM) treatment were monitored for 18 h under microscope.

## Data Availability

The data generated or analyzed during this study are included in this published article and its additional information files.

## Abbreviations

TNBC: Triple-negative breast cancer
FAC: Ferric ammonium citrate
DFO: Deferoxamine
DFP: Deferiprone
ROS: Reactive oxygen species
MDA: Malondialdehyde
LDH: Lactate dehydrogenase
PI: Propidium iodide
4-HNE: 4-Hydroxynonenal
