# Supplementary material for: Hinokitiol-iron complex is a ferroptosis inducer to inhibit triple-negative breast tumor growth
Source: Cell Biosci. 2023 May 13;13:87. doi: 10.1186/s13578-023-01044-0 (PMC10182687; doi:10.1186/s13578-023-01044-0)

**Additional figure and video captions**

**Additional Figure 1. Effect of hinokitiol (Hino) on the expression of iron-related genes in mouse tri-negative tumor cells 4T-1.** Ndufs1, SdhB, and Uqcrfs1 are Fe-S proteins as one of the subunits of complex I/II/III, respectively. IscU is a scaffold protein for Fe-S biosynthesis. TfR1: transferrin receptor 1. 4T-1 cells were treated with Hino for 24 h.

**Additional Figure 2. DFP inhibits the Hino effect on cell viability of MDA-MB-231.** Cell viability of MDA-MB-231 treated with Hino (100 µM) alone or co-treated with Hino (100 µM) and DFP (50 µM) for 24 h. DFP: Deferiprone. *, *p*< 0.05.

**Additional Figure 3. Hino functions as an iron chelator resulting in decreases of aconitase activity, protein expression of ETC complexes, and mitochondrial membrane potential in cells.** (A) The expression of NDUFS1, SDHB, UQCRFS1 and activities of mitochondrial aconitase (m-aco) and cytosolic aconitase (c-aco) in MDA-MB-231 cells treated with Hino (100 µM) or/and z-VAD-FMK (50 µM) for 24 h. (B-C) The MMP levels and ATP levels in MDA-MB-231 cells treated with Hino (100 µM) or/and z-VAD-FMK (50 µM) for 24 h. *, *p*<0.05.

**Additional Figure 4. Fe(hino)_3_ induces ferroptosis and addition of iron aggravates the effects in various tumor cells.** (A) Cell viability of human gastric cancer cell lines (BGC823, SGC7901, MGC803) after cotreatment with Hino (10 µM) and different concentrations of FAC for 24 h. (B) Cell viability after treatment with Fe(hino)_3_ (3 µM) alone or plus FAC (6 µM) for 24 h in human gastric cancer cell lines, breast cancer cells (MCF-7), and myelogenous leukemia cells (K562). (C) LDH release of BGC823 cells after 24-h incubation with Fe(hino)_3_ (5 µM) or/and Trolox (200 µM). (D) The lipid ROS in BGC823 cells treated with Fe(hino)_3_ (5 µM) alone or with Trolox (200 µM) together for 24 h, detected by flow cytometry with BODIPY-C11. (E) The lipid ROS in K562 cells treated with Fe(hino)_3_ (5 µM) alone or with Trolox (200 µM) together for 24 h. (F) Cell viability and LDH release of BGC823 cells after 24-h incubation with Fe(hino)_3_ (5 µM) or/and DFO (50 µM) or DFP (50 µM). *, *p* < 0.05; **, *p* < 0.01; ***, *p* < 0.001.

**Additional Figure 5. Fe(hino)_3_ releases iron that is efficiently chelated by DFP or DFO under cellular condition.** (A) The color of BGC823 cell pellets treated with Fe(hino)_3_ (10 µM) or/and DFO (50 µM) or DFP (50 µM) for 12 h. (B) Fe(hino)_3_ was diluted in the cell culture medium (DMEM+10% FBS) with a final concentration of 10 µM. DFO (final con. 50 µM) or DFP (final con. 50 µM) was added. After incubation at 37°C for 8 h, the samples were analyzed by UVI spectrum.

**Additional Figure 6. The quantification for Figure 5E and 5F.** The quantification of DAB-enhanced Perl’s stain positive area (A), of immunohistochemistry to present the percentage of 4-HNE positive area (B) and Ki-67 positive cells (C) in tumor tissue of Vehicle or Fe(hino)_3_ group in Figure 5E. (D) The quantification of the lung metastasis (tumor area/total lung tissue area) for Figure 5F. *, *p* < 0.05; **, *p* < 0.01; ****, *p* < 0.0001.

**Additional Figure 7. Fe(hino)_3_ treatment shows no significant toxicity in mice.** (A-C) Mice were intraperitoneally injected with Hino (5 mg/kg) or Fe(hino)_3_ (2 mg/kg). The same volume of an aqueous solution containing 5% DMSO and 2% Tween-80 as a vehicle indicated. (A) The body weight, (B) colon length, and (C) spleen volume of the mice. (D-E) Biochemical indicators after Fe(hino)_3_ (2 mg/kg) treatment. 4T-1 cells were orthotopically injected into the mammary pad of female Balb/c mice. (D) No obvious side effects on the liver, kidney, and heart function in the Fe(hino)_3_ group except creatinine with mild and significant decrease. (E) The body weight of the mice harboring tumors, recorded every other day. *, *p* < 0.05.

**Additional Video 1. The morphological changes of MDA-MB-231 cells after Fe(hino)_3_ treatment.** The morphological changes of MDA-MB-231 cells after Fe(hino)_3_ (5 µM) treatment were monitored for 18 h under microscope.


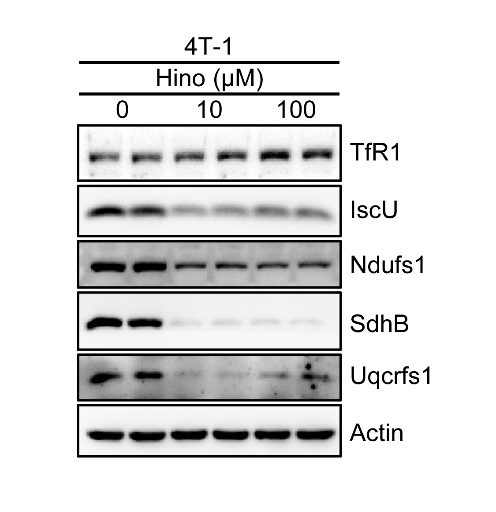
**Additional Figure 1**

**Additional Figure 2**


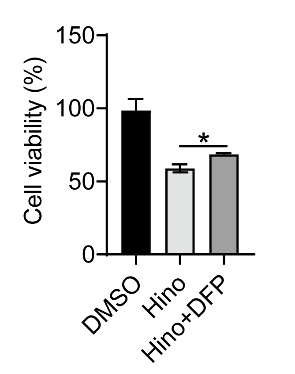


**Additional Figure 3**


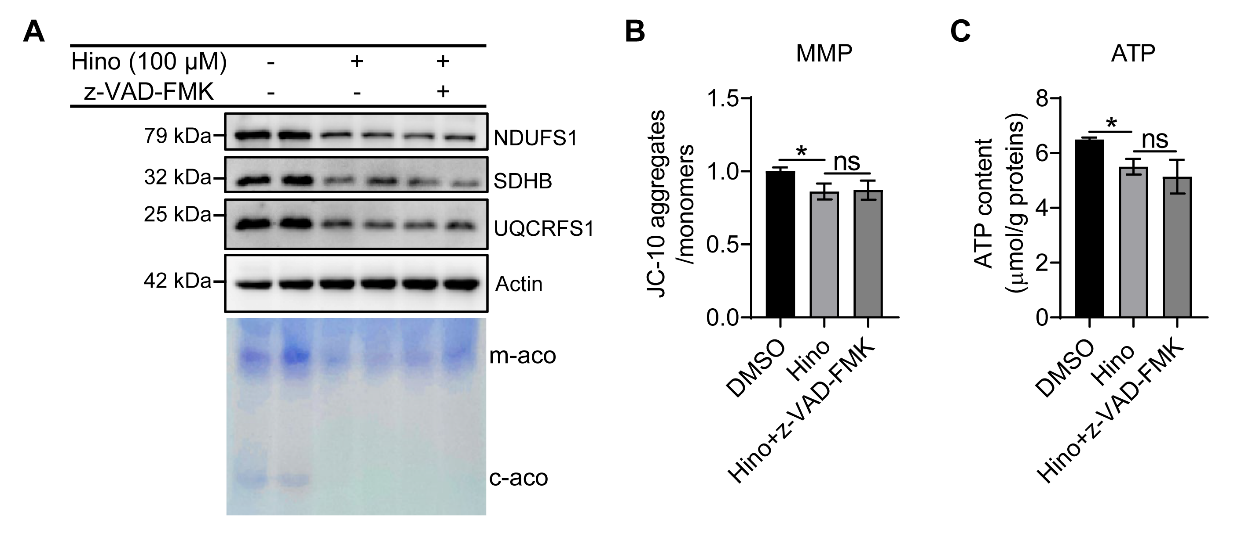


**Additional Figure 4**


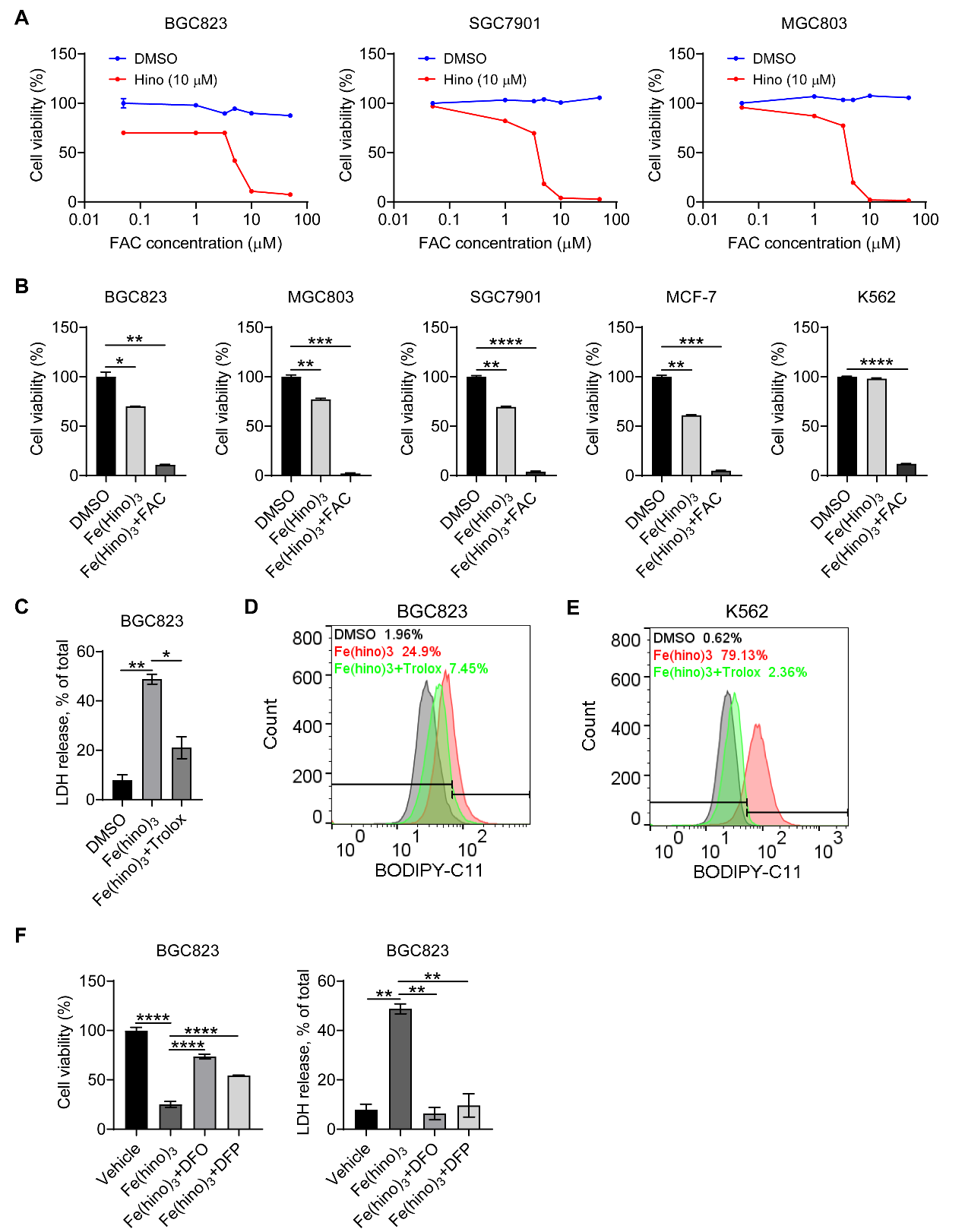


**Additional Figure 5**


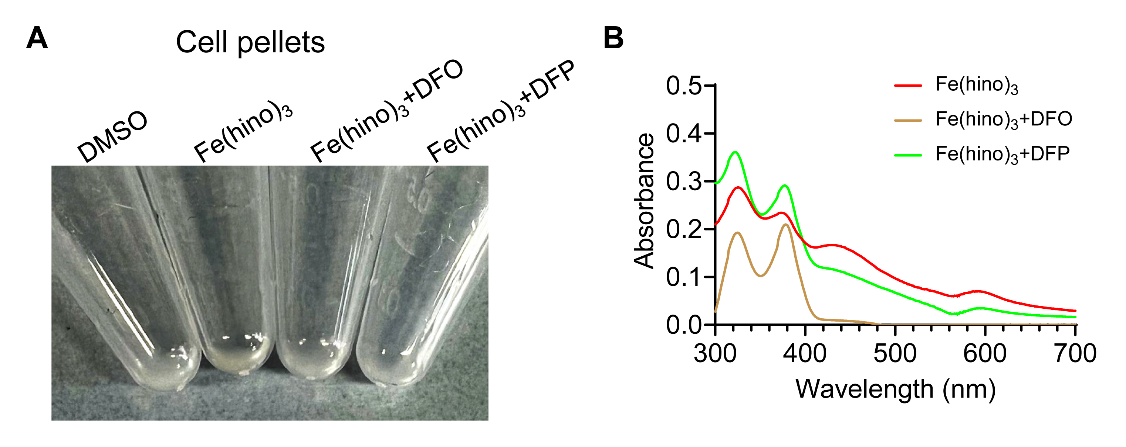


**Additional Figure 6**


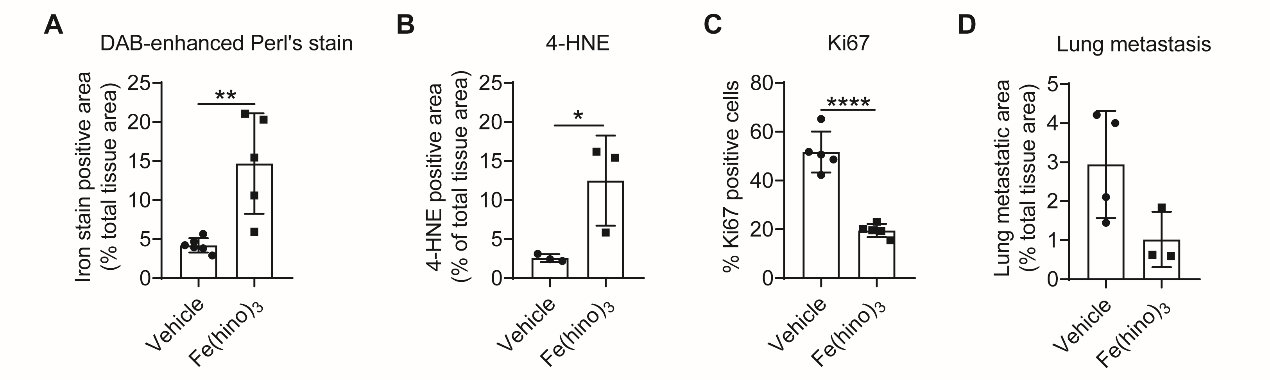


**Additional Figure 7**


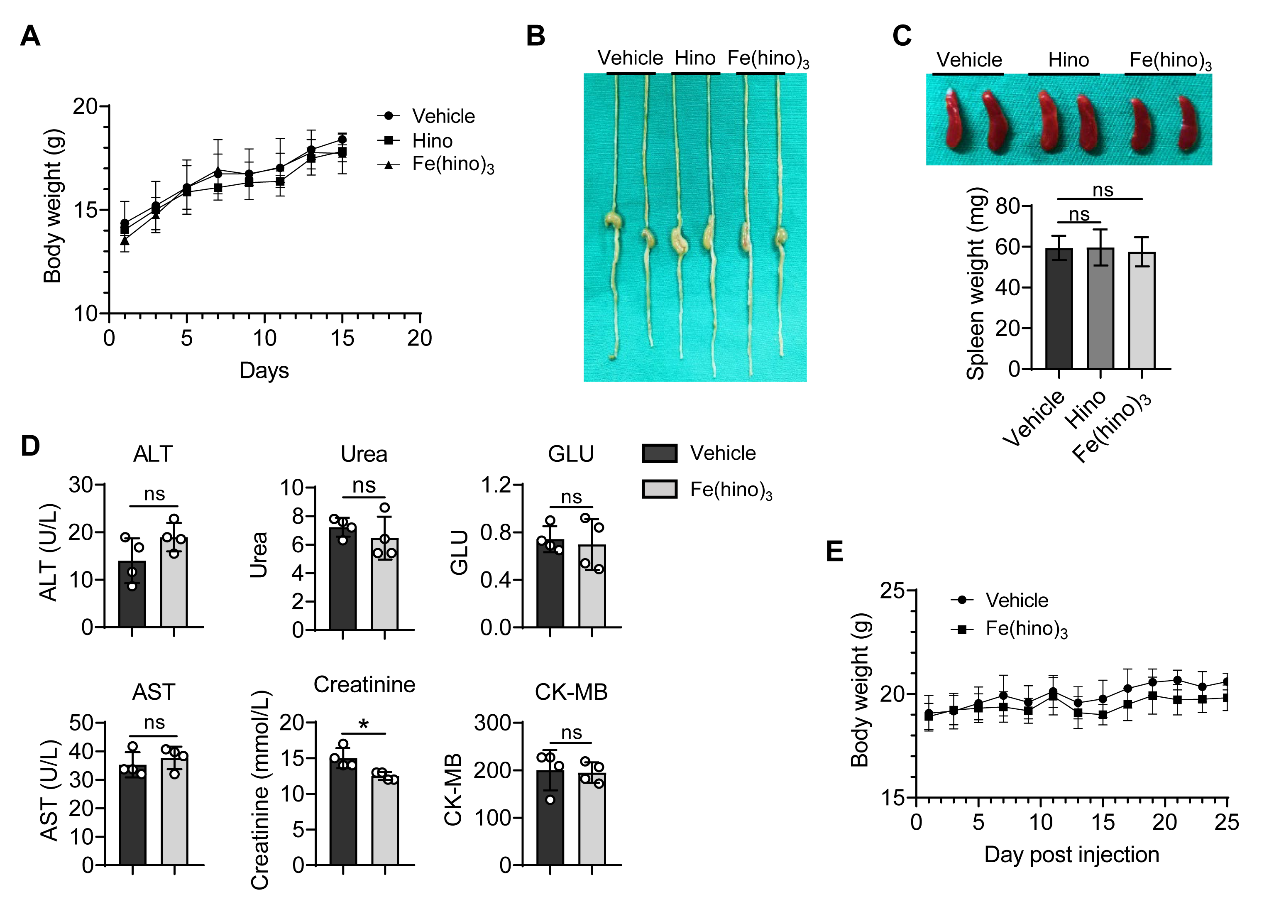

Supplement: Supplementary file 1 — Additional file 1: Figure S1. Effect of hinokitiol (Hino) on the expression of iron-related genes in mouse tri-negative tumor cells 4T-1. Ndufs1, SdhB, and Uqcrfs1 are Fe-S proteins as one of the subunits of complex I/II/III, respectively. IscU is a scaffold protein for Fe-S biosynthesis. TfR1: transferrin receptor 1. 4T-1 cells were treated with Hino for 24 h. Figure S2. DFP inhibits the Hino effect on cell viability of MDA-MB-231. Cell viability of MDA-MB-231 treated with Hino (100 µM) alone or co-treated with Hino (100 µM) and DFP (50 µM) for 24 h. DFP: Deferiprone. *, p < 0.05. Figure S3. Hino functions as an iron chelator resulting in decreases of aconitase activity, protein expression of ETC complexes, and mitochondrial membrane potential in cells. (A) The expression of NDUFS1, SDHB, UQCRFS1 and activities of mitochondrial aconitase (m-aco) and cytosolic aconitase (c-aco) in MDA-MB-231 cells treated with Hino (100 µM) or/and z-VAD-FMK (50 µM) for 24 h. (B-C) The MMP levels and ATP levels in MDA-MB-231 cells treated with Hino (100 µM) or/and z-VAD-FMK (50 µM) for 24 h. *, p<0.05. Figure S4. Fe(hino)3 induces ferroptosis and addition of iron aggravates the effects in various tumor cells. (A) Cell viability of human gastric cancer cell lines (BGC823, SGC7901, MGC803) after cotreatment with Hino (10 µM) and different concentrations of FAC for 24 h. (B) Cell viability after treatment with Fe(hino)3 (3 µM) alone or plus FAC (6 µM) for 24 h in human gastric cancer cell lines, breast cancer cells (MCF-7), and myelogenous leukemia cells (K562). (C) LDH release of BGC823 cells after 24-h incubation with Fe(hino)3 (5 µM) or/and Trolox (200 µM). (D) The lipid ROS in BGC823 cells treated with Fe(hino)3 (5 µM) alone or withTrolox (200 µM) together for 24 h, detected by flow cytometry with BODIPY-C11.(E) The lipid ROS in K562 cells treated with Fe(hino)3 (5 µM) alone or with Trolox (200 µM) together for 24 h. (F) Cell viability and LDH release of BGC823 cells after 24-h incu [file 13578_2023_1044_MOESM1_ESM.docx]
